# Celiac axis stenosis due to median arcuate ligament compression in a patient who underwent pancreatoduodenectomy; intraoperative assessment of hepatic arterial flow using Doppler ultrasonography: a case report

**DOI:** 10.1186/s13256-018-1614-2

**Published:** 2018-04-11

**Authors:** Masateru Yamamoto, Toshiyuki Itamoto, Akihiko Oshita, Yasuhiro Matsugu

**Affiliations:** 1 Department of Gastroenterological, Breast and Transplant Surgery, Hiroshima Prefectural Hospital, Hiroshima, Japan; 2 Department of Gastroenterological and Transplant Surgery, Applied Life Science, Institute of Biomedical and Health Science, Hiroshima University, Hiroshima, Japan

**Keywords:** Celiac axis stenosis, Doppler ultrasonography, Median arcuate ligament, Pancreatoduodenectomy, Hepatic arterial flow, Resistive index

## Abstract

**Background:**

Celiac axis stenosis due to compression by the median arcuate ligament has been reported in patients undergoing pancreaticoduodenectomy; it leads to the development of major collateral pathways that feed the hepatic artery. Dividing these important collaterals during pancreaticoduodenectomy can cause ischemic complications which may lead to a high mortality rate. To prevent these complications, it is necessary to assess intrahepatic arterial flow.

**Case presentation:**

A 71-year-old Japanese man with anorexia was referred to us for the treatment of alcoholic chronic pancreatitis. Computed tomography revealed a pancreatic head tumor with a calculus, associated with the dilatation of the main pancreatic duct and intrahepatic bile duct. Three-dimensional imaging demonstrated focal narrowing in the proximal celiac axis due to median arcuate ligament compression and a prominent gastroduodenal artery that fed the common hepatic artery. The preoperative diagnosis was alcoholic chronic pancreatitis with common bile duct obstruction and celiac axis stenosis due to median arcuate ligament compression. Pancreaticoduodenectomy with median arcuate ligament release was scheduled. Before the division of the median arcuate ligament, the peak flow velocity and resistive index of his intrahepatic artery measured with Doppler ultrasonography decreased from 37.7 cm/second and 0.510, respectively, to 20.6 cm/second and 0.508 respectively, when his gastroduodenal artery was clamped. However, these values returned to baseline levels after the division of the median arcuate ligament. These findings suggested that pancreaticoduodenectomy could be performed safely. Our patient was discharged on postoperative day 17 without significant complications.

**Conclusion:**

The intraoperative quantitative evaluation of intrahepatic arterial blood flow using Doppler ultrasonography was useful in a patient who underwent pancreaticoduodenectomy, who had celiac axis stenosis due to compression by the median arcuate ligament.

## Background

Blood supply to the abdominal viscera mainly arises from the celiac artery and superior mesenteric artery (SMA). The pancreatic head region is the most important site of communication between these two arterial systems via the gastroduodenal artery (GDA) and the pancreaticoduodenal arcades, respectively. Pancreaticoduodenectomy (PD) involves the division of GDA and resection of the pancreaticoduodenal arcades, which depend on both GDA and SMA.

Celiac axis stenosis (CAS) caused by external compression or internal occlusion has been reported in 2 to 7.6% of patients undergoing PD [1–6]. Compression by the median arcuate ligament (MAL) is the most common cause of CAS [6]. In CAS, the division of GDA in PD can cause ischemic complications including anastomotic dehiscence, liver failure, and liver abscess. All complications lead to a high rate of mortality, especially if the collateral pathways from SMA are inadequate [6]. To prevent these serious complications, the GDA clamping test is mandatory before the division of GDA, ensuring that sufficient hepatic blood flow is preserved by ascertaining the satisfactory pulsation of the proper hepatic artery and/or by Doppler ultrasonography. Poor arterial perfusion in the liver during the clamping test necessitates MAL division with or without additional reconstruction of the celiac artery to restore blood flow to the liver. To the best of our knowledge, no reports have described quantitative criteria for hepatic blood flow restoration using Doppler ultrasonography.

Here, we report a case of PD performed safely with quantitative intrahepatic arterial flow evaluation using intraoperative Doppler ultrasonography.

## Case presentation

**Fig. 1:**
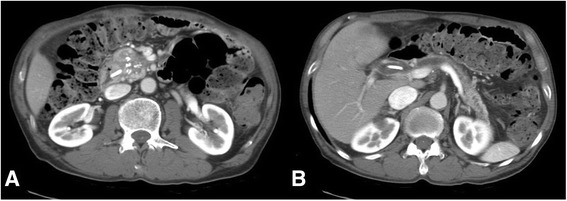
Computed tomography findings. The calculus within the swollen pancreatic head (**a**) led to the dilatation of the common bile duct, intrahepatic bile duct, and main pancreatic duct (**b**)

**Fig. 2:**
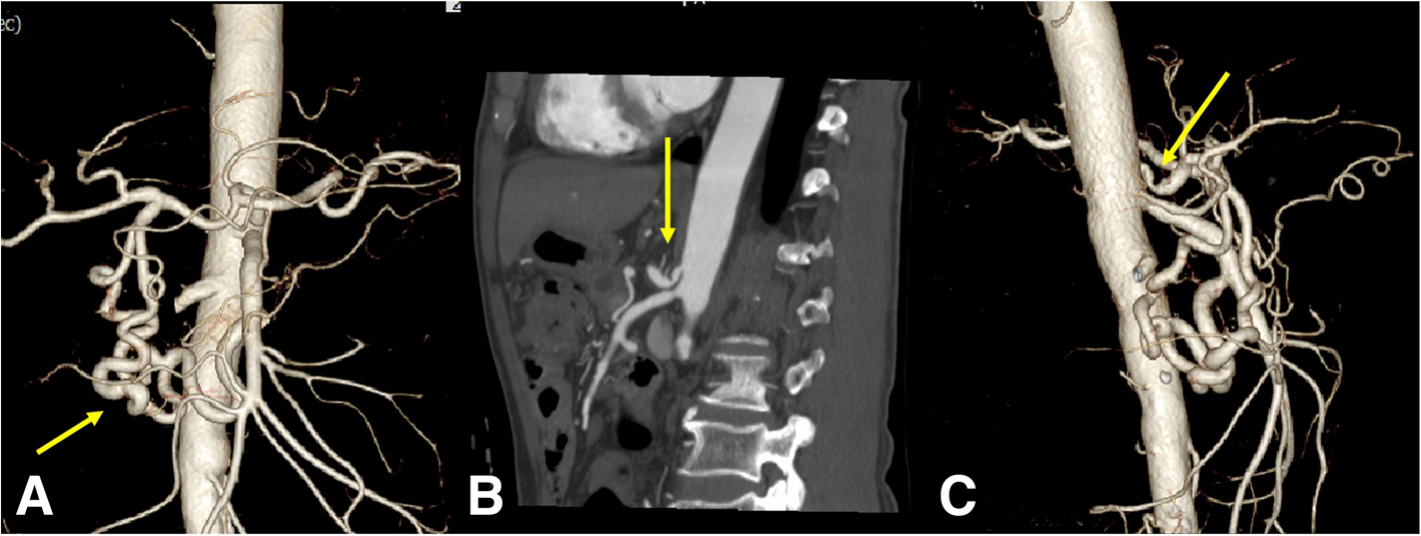
Three-dimensional volume-rendered images. The prominent gastroduodenal artery that fed the common hepatic artery from superior mesenteric artery indicated that celiac arterial flow might cease or reduce (**a**). Acute angulation and narrowing of the proximal celiac axis resulted in poststenotic dilatation, which created a “hooked” appearance (*arrow*) (**b**, **c**)

A 71-year-old Japanese man with chronic pancreatitis and pancreatic diabetes, who complained of anorexia was referred to our hospital. Laboratory findings on admission included total bilirubin, direct bilirubin, albumin, and glycated hemoglobin (hemoglobin A1c) which showed values of 1.6 mg/dL, 0.9 mg/dL, 3.5 g/dL, and 10.3%, respectively; Aspartate and alanine aminotransferase, alkaline phosphatase, and gamma-glutamyl transpeptidase showed concentrations of 63 U/L, 141 U/L, 942 U/L, and 1076 U/L, respectively. Serum carbohydrate antigen 19-9 was elevated (112 U/mL). Computed tomography (CT) showed a calculus within the swollen pancreatic head (Fig. 1a), which had caused dilatation of the common bile duct, intrahepatic bile duct, and main pancreatic duct (Fig. 1b). These findings were clarified by endoscopic retrograde cholangiopancreatography. Endoscopic ultrasonography (EUS) revealed a low echoic mass (40 mm in diameter) in the pancreatic head in which a strong echo with acoustic shadow was detected. EUS-guided fine-needle aspiration cytology for the pancreatic head tumor showed no malignancy. Three-dimensional volume-rendered imaging demonstrated a prominent GDA that fed the common hepatic artery of his SMA, indicating that celiac arterial flow might cease or reduce (Fig. 2a). A sagittal maximum-intensity projection CT angiogram and three-dimensional volume-rendered imaging demonstrated acute angulation and narrowing of the proximal celiac axis (CA) resulting in poststenotic dilatation, which created a “hooked” appearance (*arrow*) that was characteristic of MAL compression (Fig. 2b, c).

**Fig. 3:**
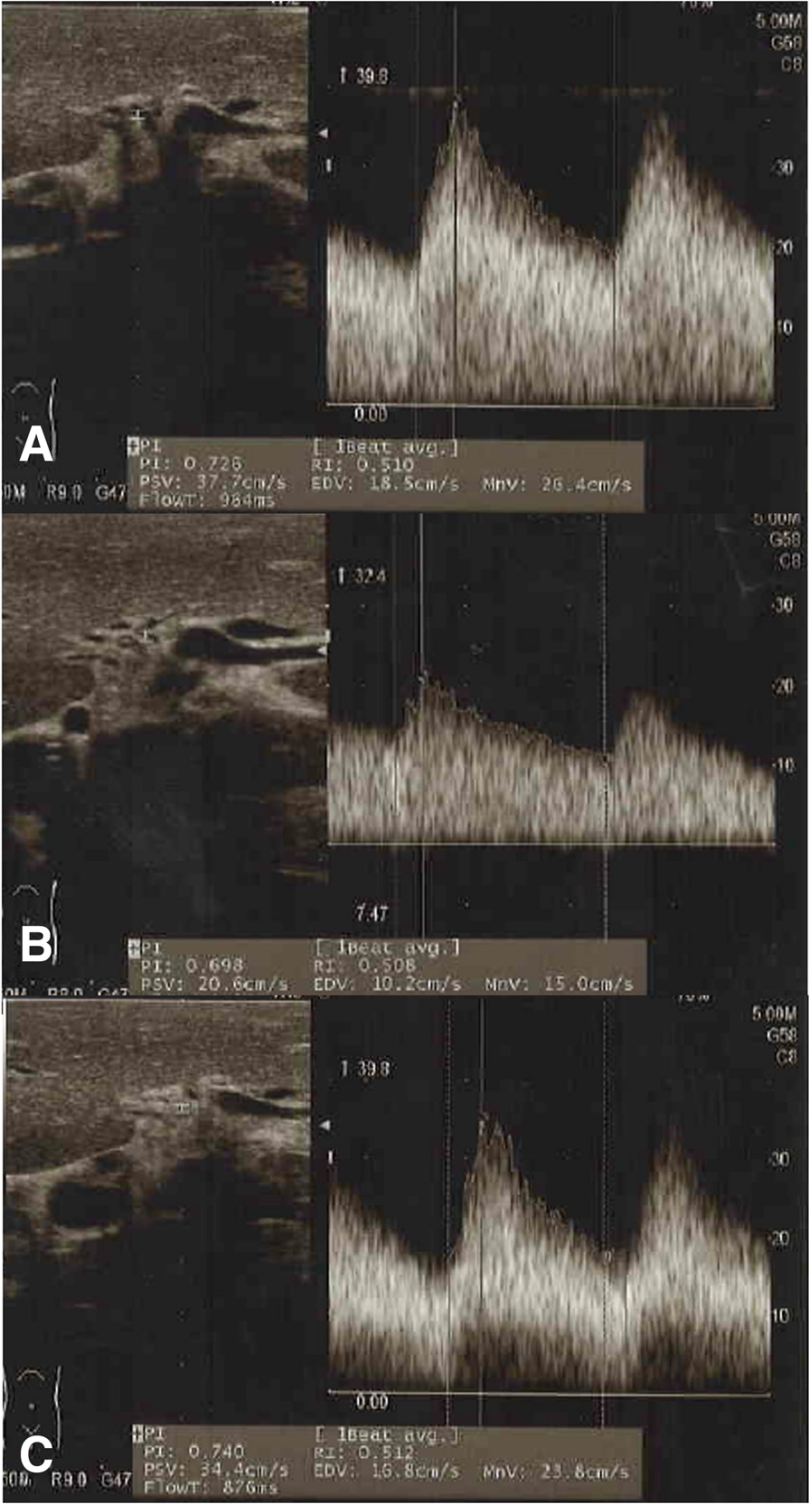
Doppler ultrasonography for gastroduodenal artery clamping test. The peak systolic and mean velocities of intrahepatic arterial flow decreased from 37.7 cm/second and 26.4 cm/second, respectively, before gastroduodenal artery clamping to 20.6 cm/second and 15.0 cm/second, respectively, after gastroduodenal artery clamping. Similarly, the resistive index decreased from 0.510 to 0.508, respectively (**a**, **b**). After the median arcuate ligament was released, the peak systolic and mean velocities of intrahepatic blood flow were restored to 34.4 cm/second and 23.8 cm/second, respectively, during gastroduodenal artery clamping. The resistive index also restored to 0.512 (**c**)

Based on these findings, the preoperative diagnosis was symptomatic mass-forming chronic pancreatitis and CAS due to MAL compression; thus, PD with or without MAL release was scheduled. The GDA clamping test was performed by measuring the intrahepatic arterial flow using Doppler ultrasonography before GDA division. Waveform did not change to tardus-parvus pattern after clamping the GDA. The peak systolic and mean velocities of the intrahepatic arterial flow decreased from 37.7 cm/second and 26.4 cm/second, respectively, before GDA clamping, to 20.6 cm/second and 15.0 cm/second, respectively, after GDA clamping. Similarly, the resistive index (RI) decreased from 0.510 to 0.508 (Fig. 3a, b). These changes were important and indicated the necessity for MAL release to minimize ischemic complications in our patient’s liver. MAL release was performed as follows. The right diaphragmatic crus was progressively divided on the right side of his abdominal aorta at the level of SMA origin. Thereafter, the right side and the upper edge of the CA were progressively freed of all dense fibrous tissue. MAL release restored the peak systolic and mean velocities of intrahepatic blood flow during the GDA clamping to 34.4 cm/second and 23.8 cm/second, respectively. RI was also restored to 0.512, enabling us to divide GDA safely and to perform a subsequent dissection of the pancreas head (Fig. 3c).

**Fig. 4:**
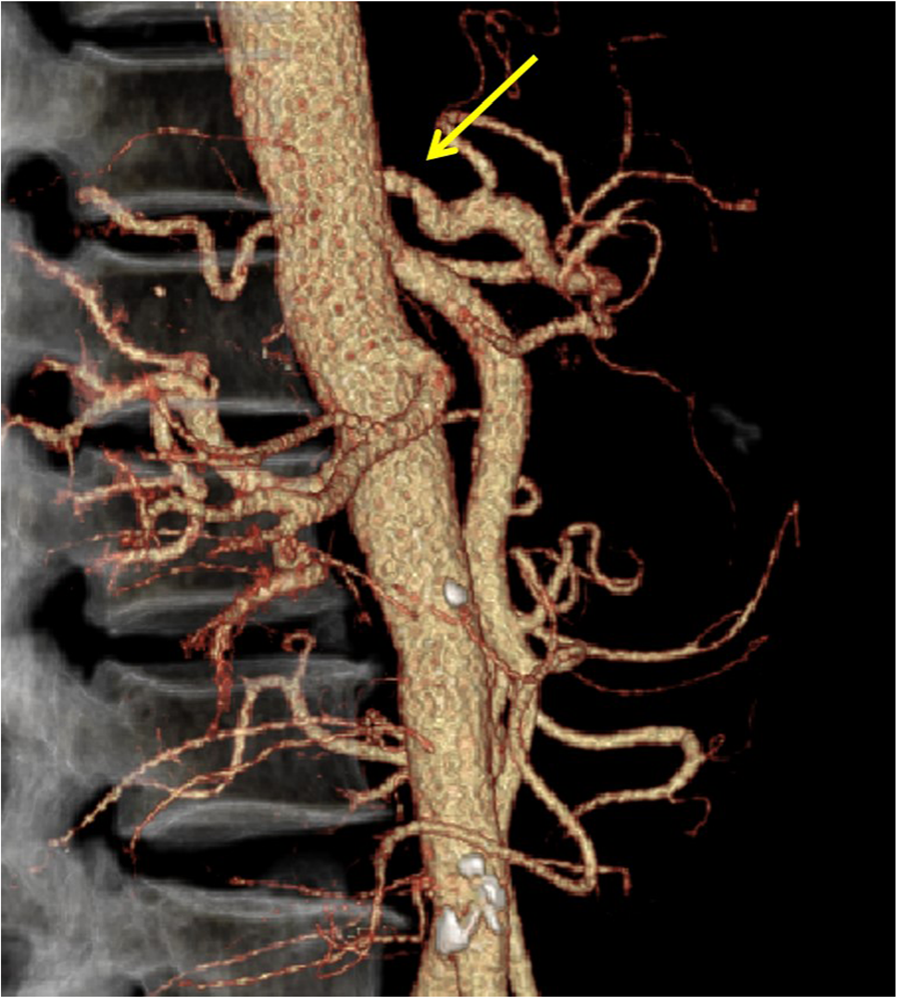
Three-dimensional volume-rendered images on postoperative day 30. There was no stenosis of the celiac artery origin (arrow)

His postoperative course was uneventful, and he was discharged on postoperative day 17. Abdominal CT on postoperative day 30 showed that there was no stenosis at the CA (Fig. 4).

## Discussion and conclusions

External compression at the origin of the CA may occur and is mainly due to an anomaly of MAL. MAL is a fibrous arch that unites the diaphragmatic crura on either side of the aortic hiatus. The ligament usually passes over the aorta and superior to the origin of the CA; however, it passes at the level of or below the origin of the CA in up to 33% of fresh autopsy specimens [7] and may lead to the symptomatic compression of the CA, that is, MAL syndrome, which was first described in 1917 [8].

It has become possible to detect uncommon abnormalities using recent imaging techniques. Three-dimensional CT angiography makes it possible to diagnose CAS caused by various etiologies with 96% sensitivity and to determine the etiology of CAS with 92% accuracy [6]. CAS caused by MAL compression often exhibits, on imaging, more than 50% stenosis approximately 5 mm distal to the celiac artery bifurcation [6]. Hooked appearance is a characteristic finding in which the origin of CA is deformed into a hook shape [9]. CAS results in the development of major collateral pathways (pancreaticoduodenal arcades or the dorsal pancreatic artery) which arise from SMA, resulting in the feeding of the branches of the common hepatic artery through retrograde flow via GDA or the arc of Buhler [10–12]. Dividing these important collaterals to the upper abdominal organs during PD in a patient with CAS can cause postoperative life-threatening ischemic complications including hepatic and anastomotic ischemia [13, 14].

To prevent these serious complications, CAS caused by MAL compression can be treated by complete MAL division during PD. However, some investigators have argued that the division of GDA during PD in patients with CAS does not always result in ischemic complications of the upper abdominal organs [1–3], reporting that only 13 to 17% of patients with CAS required arterial reconstruction during PD, because abundant collateral anastomoses other than the pancreatic head arcade might develop between the CA tributaries and SMA tributaries [1, 3]. Song *et al*. also reported that in patients with CAS, collateral pathways, such as from the dorsal pancreatic artery or replaced right hepatic artery, existed in approximately 80% of the patients [12]. However, Nara *et al*. reported that two of four patients with CAS, who did not undergo MAL division, developed liver abscesses after PD [15].

In cases of CAS, intraoperative trial clamping of GDA with the palpation of the hepatic artery or with the assessment of intrahepatic arterial flow by Doppler ultrasonography is of paramount importance before the division of GDA in order to prevent postoperative ischemic complications [3]. The weakening of hepatic arterial pulsation and the decrease of hepatic arterial flow by Doppler ultrasonography in the GDA clamping test indicate the necessity of MAL division during PD. Gaujoux *et al*. reported that CAS due to MAL was diagnosed in approximately 10% of the candidates for PD and approximately half of these, who became candidates for MAL division, were found to have hemodynamically significant CAS during the GDA clamping test via Doppler assessment [6]. However, they did not show the threshold of a significant decrease in hepatic arterial blood flow. Nara *et al*. also described the need for intraoperative assessment using Doppler ultrasonography in patients with CAS during PD [15]. Decreased or absent hepatic arterial pulsation or signal using intraoperative Doppler ultrasonography would indicate the need for vascular reconstruction or MAL release. However, there is no consensus on the threshold beyond which MAL division or an additional intervention is required.

Doppler ultrasonography can evaluate blood flow not only qualitatively but also quantitatively in the field of liver transplantation [16, 17]. The normal hepatic artery Doppler waveform shows a rapid systolic upstroke following continuous diastolic flow. The acceleration time and RI can serve as indicators of hepatic arterial blood flow assessment. The acceleration time, which represents the time from the end of diastole to the first systolic peak, should be less than 80 ms. The RI, which represents (peak systolic velocity – end diastolic velocity)/peak systolic velocity, should be between 0.5 and 0.7 [16]. Tardus-parvus pattern of waveform with an acceleration time greater than 80 ms and RI less than 0.5 represent insufficient arterial flow due to hepatic artery stenosis in the setting of liver transplantation [17].

In the present case when GDA was clamped, the peak and mean velocities and RI decreased reproducibly compared with their baseline levels which were measured before clamping, although RI was maintained at more than 0.50 and the tardus-parvus pattern of waveform was not found. These decreased values almost returned to the baseline levels during GDA clamping just after MAL division.

To establish definitive criteria and to clarify the threshold for MAL division or for additional hepatic arterial reconstruction during PD for patients with CAS, a large number of patients should be studied based on the precise evaluation of the velocity of hepatic arterial inflow using Doppler ultrasonography. However, MAL division might be indicated when a reproducible decrease in hepatic arterial flow is noted during GDA clamping. Moreover, restoration of flow values to baseline levels by MAL division might imply the unnecessariness of additional hepatic arterial reconstruction.

## Abbreviations

CA: Celiac axis
CAS: Celiac axis stenosis
CT: Computed tomography
EUS: Endoscopic ultrasonography
GDA: Gastroduodenal artery
MAL: Median arcuate ligament
PD: Pancreaticoduodenectomy
RI: Resistive index
SMA: Superior mesenteric artery
